# Consistent Intraocular Pressure Reduction by Solid Drug Nanoparticles in Fixed Combinations for Glaucoma Therapy

**DOI:** 10.1002/advs.202401648

**Published:** 2024-06-14

**Authors:** Da Huang, Pedro Norat, Lin Qi, Anna Chernatynskaya, James D. Cole, Vimalin Jeyalatha Mani, Lei Xu, Xiaorong Liu, Hu Yang

**Affiliations:** ^1^ College of Biological Science and Engineering Fuzhou University Fuzhou Fujian 350108 China; ^2^ Linda and Bipin Doshi Department of Chemical and Biochemical Engineering Missouri University of Science and Technology Rolla MO 65409 USA; ^3^ Department of Biology University of Virginia Charlottesville VA 22904 USA; ^4^ Department of Psychology University of Virginia Charlottesville VA 22904 USA; ^5^ Program in Fundamental Neuroscience University of Virginia Charlottesville VA 22904 USA; ^6^ Present address: Department of Psychology West Virginia University Morgantown WV 26506 USA

**Keywords:** anti‐glaucoma drugs, cornea permeation, ocular hypertension, scalable fabrication, solid drug nanoparticles

## Abstract

Efficient topical drug delivery remains a significant challenge in glaucoma management. Although nanoparticle formulations offer considerable promise, their complex preparation processes, co‐delivery issues, and batch consistency have hindered their potential. A scalable fabrication strategy is developed here for preparing solid drug nanoparticles (SDNs) with enhanced drug delivery efficiency. Utilizing hydrophobic antiglaucoma drugs brimonidine (BM) and betaxolol (BX), uniform fixed combination BM/BX SDNs are fabricated through a continuous process, improving batch‐to‐batch consistency for combined glaucoma treatment. With trehalose being used as a lyoprotectant, BM/BX SDNs can be stored as dry powder and easily reconstituted in phosphate buffered saline. Importantly, reconstituted BM/BX SDNs form clear, homogenous solutions, and exhibit negligible cytotoxicity and irritation, making them well‐suited for topical administration as eyedrops. Ex vivo and in vivo studies demonstrated that topically applied BM/BX SDNs permeate through the cornea significantly (about two fold to three fold) compared to their hydrophilic counterparts, i.e., brimonidine tartrate, and betaxolol hydrogen chloride. Notably, BM/BX SDNs displayed consistent intraocular pressure lowering effects in vivo in both normotensive rats and glaucoma mice. Collectively, this study demonstrates the potential of the scalable fabrication strategy and the resultant BM/BX SDNs for improving glaucoma management through eyedrops.

## Graphical abstract

Using a scalable method for fabricating solid drug nanoparticles (SDNs), SDNs in fixed combination of the hydrophobic antiglaucoma drugs brimonidine (BM) and betaxolol (BX) are consistently produced. These SDNs demonstrated enhanced ocular permeation and sustained intraocular pressure reduction, indicating their effectiveness for glaucoma therapy.

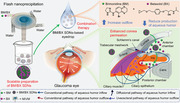

## 1 Introduction

Approximately 80 million people suffered from glaucoma globally in 2020,^[^ ^1^ ^]^ and this number is anticipated to rise to 111.8 million by 2040.^[^ ^2^ ^]^ Many glaucoma patients suffer an elevated intraocular pressure (IOP) due to a clogged or damaged drainage system. If left untreated, elevated IOP leads to retinal ganglion cell death and irreversible vision loss. Current drug or surgery treatments primarily aim to reduce or control IOP by promoting the outflow of aqueous humor and/or reducing the production of aqueous humor.^[^ ^3^, ^4^, ^5^ ^]^ The first‐line drugs were designed to reduce aqueous humor production using beta blockers (e.g., betaxolol (BX), timolol), adrenergic agonists (e.g., brimonidine (BM)), or carbonic anhydrase inhibitors (e.g., dorzolamide) and/or promote the outflow of aqueous humor via the uveoscleral pathway using prostaglandins.^[^ ^6^, ^7^ ^]^

Among different delivery strategies, topical administration of antiglaucoma medications is preferred because of its minimal invasiveness. However, many drugs are hydrophobic, hence their salt counterparts with enhanced solubility in water are used to prepare eye drops. For example, hydrophobic BM and BX are formulated as brimonidine tartrate (BT) and betaxolol hydrochloride (BH) to increase their solubility in water. Nonetheless, their bioavailability is extremely low (<5%), a trade‐off due to the inefficient transport of hydrophilic molecules across lipophilic biological membranes and the challenge posed by precorneal tear clearance.^[^ ^8^, ^9^, ^10^, ^11^, ^12^ ^]^ This leads to frequent dosing and poor patient compliance, with many patients struggling to adhere to their regimen for more than a few weeks, as revealed in electronic monitoring studies.^[^ ^13^, ^14^ ^]^ When a single drug falls short in controlling IOP, multiple drugs with diverse therapeutic mechanisms are often prescribed for enhanced treatment outcomes. However, efficiently delivering these drugs in a single formulation remains a challenge in glaucoma management.

In the past decades, nanotechnology has revolutionized the formulation of hydrophobic drugs because nanoparticle formulations enable excellent dispersity of hydrophobic drugs in aqueous solutions and allow them to pass through lipophilic barriers via paracellular transport and transcytosis. Two common strategies are employed to develop nanoparticle formulations: utilizing nanocarriers such as polymeric micelles, liposomes, and nanogels; or fabricating solid drug nanoparticles (SDNs), where each nanoparticle only consists of drugs.^[^ ^15^ ^]^ In comparison to nanocarriers, SDNs exhibit higher drug loading, more flexibility in integrating different drugs in one formulation, and the carrier‐free nature eliminates extra cost and safety concerns aroused by the carriers.^[^ ^16^, ^17^ ^]^ SDNs are normally prepared via nanomilling method, which has been successful in development of several medicines such as Theodur, Rapamune, TriCor, and Emend.^[^ ^18^ ^]^ However, nanomilling is not suitable for antiglaucoma drugs due to their amorphous, hydrolytically sensitive, or low melting point nature. Nanoprecipitation, which involves adding drops of drug solution in water miscible organic solvent to water or aqueous solution, has been adopted to develop SDNs. Yet nanoparticles formed by nanoprecipitation have a broad size range and poor consistency from batch to batch,^[^ ^19^ ^]^ limiting industrial production and clinical translation.

**Figure 1:**
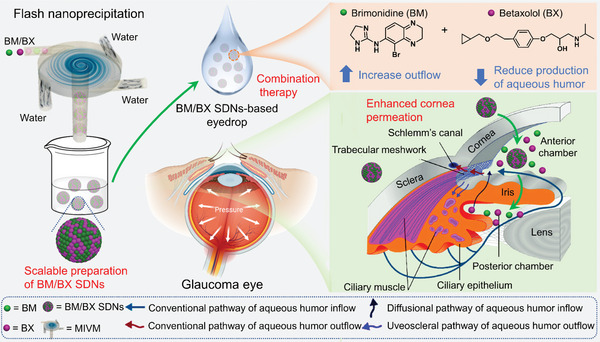
Schematic illustration of the fabrication of BM/BX SDNs via flash nanoprecipitation and their topical use for glaucoma therapy.

To address these issues, considerable efforts have been devoted to develop new production methods that allow scaling up preparation of uniform SDNs. In 2008, Prud'homme et al. designed a multi‐inlet vortex mixer (MIVM) for the continuous production of polymeric micelles by flash nanoprecipitation.^[^ ^20^ ^]^ This MIVM‐based flash nanoprecipitation produced SDNs of methotrexate showed a narrow‐size distribution and batch‐to‐batch consistency.^[^ ^21^ ^]^ In this study, the nanoprecipitation method was successfully applied to create SDNs from various antiglaucoma drugs on a larger scale. SDNs containing two hydrophobic antiglaucoma drugs BM and BX were prepared using the flash nanoprecipitation method and their bioavailability and antiglaucoma effects ex vivo and in vivo were tested (**Figure** **1**). The new formulations were examined in vivo in both normotensive rats and glaucoma mice. In particular, glaucoma mice used in the study are a genetic mouse model of open‐angle glaucoma,^[^ ^22^, ^23^, ^24^ ^]^ *Angiopoietin1* conditional knockout mice (A1 cKO).^[^ ^10^, ^22^, ^23^, ^24^ ^]^

## 2 Results

### 2.1 Fixed Combination SDNs Exhibit Excellent Batch Consistency and Stability Following Reconstitution

**Figure 2:**
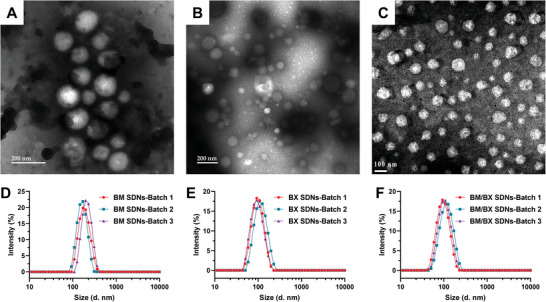
SDNs exhibit good batch consistency. A–C) Representative TEM image of BM SDNs (A), BX SDNs (B), and BM/BX SDNs (C). D–F) Size distributions of BM SDNs (D), BX SDNs (E), and BM/BX SDNs (F). Each color represents the results from one batch.

As shown in Figure S1 (Supporting Information), the MIVM‐based nanoparticle generation system consists of an MIVM and syringe pumps. The MIVM has four inlets for introducing different components, and vortex mixing of antiglaucoma drugs in methanol with water in the inner small chamber generates SDNs via nanoprecipitation. To evaluate the robustness of the proposed fabrication method, different antiglaucoma drugs, including BM, BX, and a mixture of them, were employed to prepare SDNs. SDNs composed of only BM or BX (named BM SDNs and BX SDNs) were prepared, and the representative TEM images show they are uniform, spherical particles with an average size of ≈150 and 80 nm, respectively (**Figure** **2**A,B). The difference in size between BM SDNs and BX SDNs is attributed to their varying levels of hydrophobicity, which are directly related to the distinct structural characteristics of each compound. BM/BX SDNs with a BM/BX mass ratio of 2/5 was obtained using the same method. Their morphology looks similar to that of BX SDNs (Figure 2C), as BX accounts for a higher proportion in the mixture. The hydrodynamic sizes of these SDNs determined by DLS are slightly higher than those revealed by TEM (Figure 2D–F), which is reasonable because the particles observed under TEM were in dry state. Considering that adrenergic agonists and beta blockers are being widely used for the combination therapy of glaucoma, the BM/BX SDNs were used in the following evaluations.

**Figure 3:**
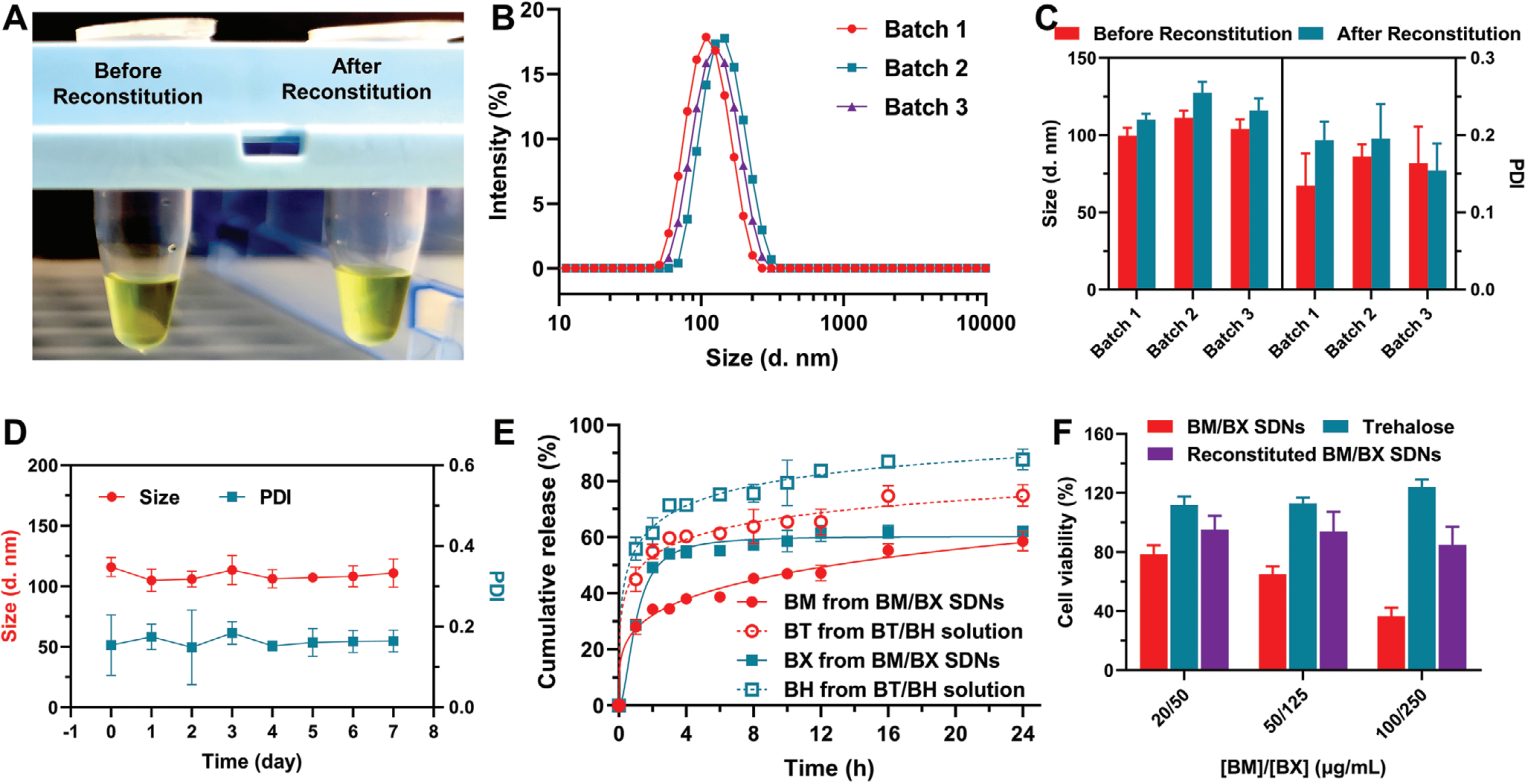
The reconstituted BM/BX SDNs exhibit a transparent appearance, good batch consistency and stability, a sustained release profile, and improved cytocompatibility. A) Appearance of BM/BX SDNs solution before and after freeze‐dry and reconstitution with trehalose as cryoprotectant; B) Size distributions of reconstituted BM/BX SDNs from different batches; C) Comparison of hydrodynamic size and PDI of the BM/BX SDNs from different batches before and after freeze‐dry and reconstitution; D) Variation of hydrodynamic size and PDI of the reconstituted BM/BX SDNs after being stored at 4 °C for different days; E) In vitro release of BM and BX from BM/BX SDNs, hydrophilic counterparts BT and BH were used as control; F) Cytotoxicity of BM/BX SDNs with or without trehalose against HCE‐2 cells. Data were expressed as mean ± SD (n = 3 for C‐E, n = 5 for F).

BM/BX SDNs were freeze‐dried in the presence of lyoprotectant trehalose and studied subsequently for their reconstitution. As shown in **Figure** **3**A, the reconstituted BM/BX SDNs solution is homogeneous and nearly transparent, showing no difference with the BM/BX SDNs solution between before and after lyophilization, confirming that BM/BX SDNs can be stored in a freeze‐dried powder form. The size distribution of reconstituted BM/BX SDNs from three different batches all showed unimodal curve (Figure 3B), and the hydrodynamic sizes only increased slightly with negligible variation in PDIs (Figure 3C), indicating that no aggregations were formed during the lyophilization.

To investigate the colloidal stability of the reconstituted BM/BX SDNs, the reconstituted solution was kept in refrigerator and the size distribution was monitored for 7 days. As summarized in Figure 3D and Figure S2 (Supporting Information), the size distribution, average hydrodynamic and PDI of the reconstituted BM/BX SDNs remained unchanged during storage.

### 2.2 BM/BX SDNs Enable Sustained Drug Release and Demonstrate Improved Cytocompatibility

Because the BM/BX SDNs are composed of hydrophobic drugs, the release is expected to slow down in comparison to the hydrophilic BT and BH. To verify our speculation, in vitro drug release experiments of BM/BX SDNs and BT/BH were carried out at 37 °C in phosphate buffer with a pH of 7.4 at sink condition. As shown in Figure 3E, more than 60% of BT and 70% of BH were released within 4 h, while less than 40% of BM and 55% of BX were released from the BM/BX SDNs after 4 h. The results demonstrated that the BM/BX SDNs allow longer release period, which enables prolonged action time of the antiglaucoma drugs.

To evaluate the biocompatibility of the BM/BX SDNs, we investigated their cytotoxicity using HCE‐2 cells. The commercially available BT and BH eyedrop contains 2 and 5 mg mL^−1^ of BT and BH, respectively. Given that most of the administered drugs are washed away by tear and the absorbed drugs will be further diluted by aqueous humor, we only evaluated the cytotoxicity of formulations with BM at a concentration of 0.02–0.1 mg mL^−1^ and BX at a concentration of 0.05–0.25 mg mL^−1^. In the tested range, the freshly prepared BM/BX SDNs showed concentration‐dependent toxicity to the cells, revealed by less than 40% viability at the highest concentration. We also noticed that the viability of cells treated with reconstituted BM/BX SDNs, was higher than 85% (Figure 3F), which may be due to the improved cytocompatibility by trehalose.^[^ ^25^, ^26^, ^27^ ^]^

### 2.3 BM/BX SDNs do not Induce Ocular Irritation

**Figure 4:**
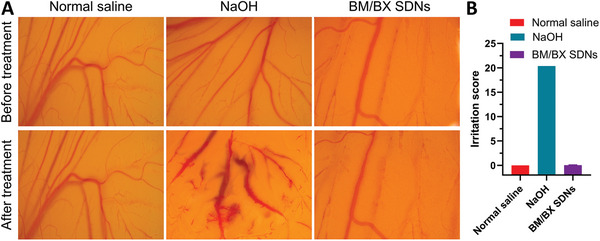
The BM/BX SDNs exhibit no ocular irritation. A) Representative photos of the CAM before and after treatment with PBS (negative control), 0.1 N NaOH (positive control) and BM/BX SDNs; B) Irritation scores of the CAM after different treatments (The error bars for Normal saline group and NaOH group are very small. Data were expressed as mean ± SD, n = 3).

The Hen's egg‐Chorioallantoic Membrane (HET‐CAM) test was applied to assess the ocular tolerability and irritation potential of the BM/BX SDNs. We observed the effects by directly applying the BM/BX SDNs onto the CAM, and these effects were then compared with those of the control groups. Specifically, normal saline served as the negative control, and 0.1 n NaOH functioned as the positive control. As exhibited in **Figure** **4**, the application of 0.1 n NaOH caused immediate hemorrhage and disintegration of blood vessels, resulting in an irritation score of 20.37. In contrast, the application of normal saline induced minimal irritation, with an irritation score of 0. Notably, the BM/BX SDNs exhibited no signs of irritation; as evidenced by the absence of observable damage or morphological changes in the CAM's blood vessels, culminating in a mean irritation score of just 0.03. These findings suggest that the BM/BX SDNs are non‐irritating and are well‐tolerated upon application.

### 2.4 SDNs Enhance the Permeation of the Formulated Drugs

**Figure 5:**
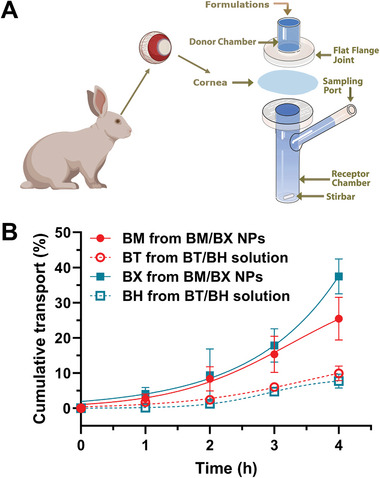
SDNs improve the ex vivo permeation of the formulated drugs across the cornea. A) Apparatus setup for ex vivo cornea permeation; B) Ex vivo permeation of BM/BX SDNs across the cornea where BT and BH free drug solutions were used as control. Data were expressed as mean ± SD (n = 3).

To investigate if the BM/BX SDNs will show improved cornea permeation, we performed ex vivo cornea permeation using a Franz Cell, with freshly excised rabbit cornea placed between the donor chamber and receptor chamber (**Figure** **5**A). Given the short retention time of topically administered eyedrops on the surface of eye, the permeation was monitored for 4 h. As summarized in Figure 5B, the BM/BX SDNs brought more drugs across the cornea. Within 4 h, the BM/BX SDNs enabled more than 30% of BM and BX to pass through the cornea, while only less than 10% of the hydrophilic counterparts BT and BH penetrated across the cornea.

**Figure 6:**
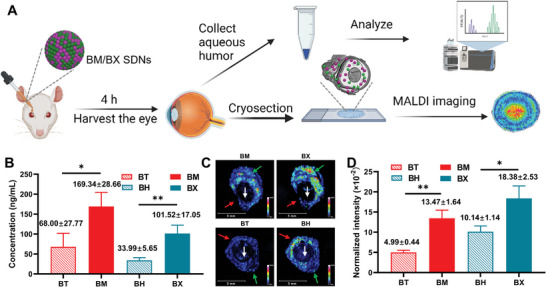
The BM/BX SDNs enhance the bioavailability of the drugs in vivo. A) Schematic illustration of the procedures for in vivo permeation and analysis; B) Drug concentrations detected in the aqueous humor of rats by LC‐MS after treated with BM/BX SDNs and BT/BH solutions; C) Abundance of drugs in the rat eyes revealed by MALDI‐IMS (white arrow: lens, red arrow: cornea, green arrow: posterior part of eye); D) Quantitative analysis of the abundance of drugs in the rat eyes. Statistical analysis was performed by unpaired Student's *t*‐test (two‐tailed), and data were expressed as mean ± SD (n = 3), ^*^ *p* < 0.05, ^**^ *p* < 0.01.

To further confirm that the SDN formulation can enhance the ocular permeation of drugs, in vivo permeation experiments were carried out on rats. After 4 h of topical administration, the drugs accumulated in the aqueous humor and eye tissues were measured by LC‐MS and MALDI‐IMS, respectively (**Figure** **6**A). With the assistance of the BM/BX SDNs, the detected concentration of BM and BX in aqueous humor were about 3 times higher than that of BT and BH (Figure 6B), indicating significant enhancement of ocular permeation resulted from the BM/BX SDNs. As displayed in Figure 6C, the intensities of BM and BX in the eye sections excised from BM/BX SDNs treated eyes were much higher than those treated with BT/BH. Further quantitative analysis revealed that the abundance of BM was almost threefold higher than BT and the abundance of BX was ≈1.8‐fold higher than BH (Figure 6D), in accordance with the concentrations in the aqueous humors.

### 2.5 BM/BX SDNs Exert a Pronounced IOP Lowering Effect in Normotensive Rats

We conducted in vivo experiments using normal rats to examine the IOP‐lowering effect of BM/BX SDNs. To determine the action time of the formulations, IOP was monitored after one dose. At 6 h post‐administration, the BM/BX SDNs and BT/BH showed similar effect, but the IOP in the eyes treated with BT/BH trended to go back to the baseline at 30 h post‐treatment, while a significant IOP lowering effect for 120 h was observed in the eyes treated with the BM/BX SDNs (Figure S3A, Supporting Information). Throughout the monitoring period, the average IOP reduction caused by the BM/BX SDNs was 2.91 mmHg, which is 3.3‐fold stronger than IOP reduction resulted from the BT/BH (0.88 mmHg) (*p* < 0.005). Besides, at 24, 30, and 48 h post‐treatment, the IOP reduction of BM/BX SDNs showed significant difference relative to the BT/BH treated eyes. Therefore, we concluded that one dose of BM/BX SDNs can maintain effective IOP reduction for at least 48 h.

Furthermore, we examined whether the BM/BX SDNs could maintain therapeutic effect with reduced doses. In this study, rats were treated with three successive doses every 48 h and IOPs were monitored. Though both BT/BH and the BM/BX SDNs exhibited IOP lowering effect throughout the treatment, the average IOP reduction induced by the BM/BX SDNs was much higher than that of the BT/BH (3.84 vs 2.11, *p* < 0.005, Figure S3B, Supporting Information). Statistical analysis indicated that the differences in IOP reduction at most time points are statistically significant.

### 2.6 BM/BX SDNs Exert a Pronounced IOP Lowering Effect in Mice with Chronic IOP Elevation

**Figure 7:**
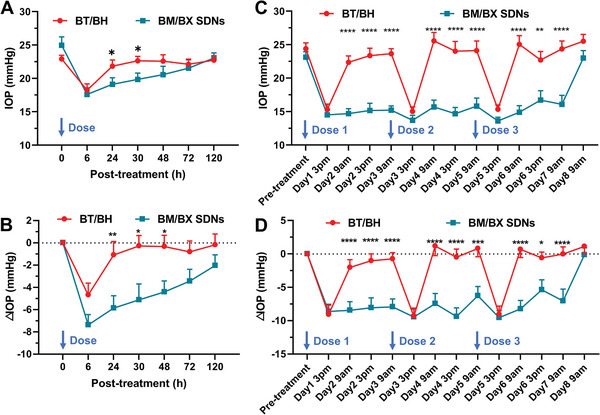
Consistent IOP reduction is achieved with the use of the BM/BX SDNs. IOP and IOP reduction of A1 cKO mice treated with A,B) a single dose or C,D) three successive doses of BT/BH or BM/BX SDNs (0.2% w/v BM, 0.5% w/v BX, 2 × 2 µL per eye). Statistical analysis was performed by unpaired Student's *t*‐test (two‐tailed), and data were expressed as mean ± SE (n = 8), ^*^ *p* < 0.05, ^**^ *p* < 0.01, ^***^ *p* < 0.005, ^****^ *p* < 0.001.

The IOP lowering effect of the BM/BX SDNs was further evaluated using A1 cKO mice as a glaucoma model. The baseline IOP of A1 cKO mice reaches ≈20 mmHg by 6 weeks of age, and stabilizes in adulthood, whereas the wild type mice usually show a IOP of ≈15 mmHg.^[^ ^10^ ^]^ After a single dose treatment, significant IOP reduction was observed for both BT/BH and BM/BX SDNs treated eyes at 6 h post‐treatment. However, the IOP of BT/BH treated group restored to baseline soon at 24 h post‐treatment. In comparison, the IOP lowering effect of the BM/BX SDNs lasted for a few days and diminished after 120 h (**Figure** **7**A,B). Over the course of the treatment, the BM/BX SDNs resulted in an average IOP reduction of 4.71 mmHg, 3.8‐fold higher than the average IOP reduction of the BT/BH group (1.24 mmHg) (*p* < 0.001).

Similarly, the IOP lowering effect of multiple doses treatment on the A1 cKO mice was investigated. It's obvious that administration of BM/BX SDNs every other day could lower the IOP significantly and then maintain it at a low level, while the treatment of BT/BH only exhibited IOP reduction at 6 h post‐treatment, then the effect vanished quickly after 24 h (Figure 7C,D).

### 2.7 BM/BX SDNs do not Alter Eye Morphology or Visual Function

**Figure 8:**
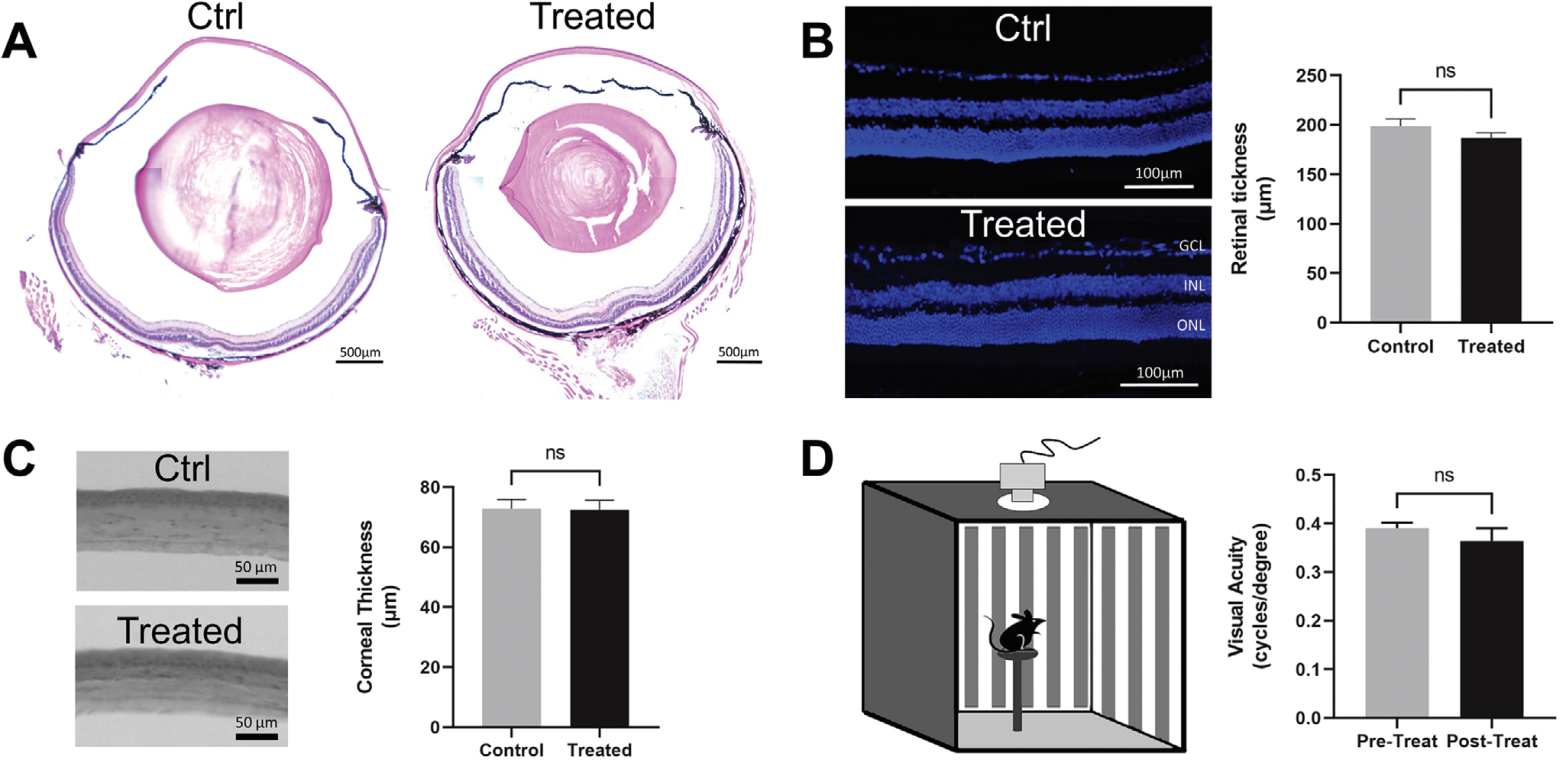
BM/BX SDNs do not alter eye morphology or visual function. A) H&E staining shows a largely normal eye morphology before and after drug treatment for 7 days; Quantifications of the thickness of B) retinal layer (n = 6) and C) cornea (n = 7). D) Optomotor test confirmed that the acuity does not change over a period of 7‐day drug treatment (n=10). Statistical analysis was performed by unpaired Student's *t*‐test (two‐tailed), and data were expressed as mean ± SE.

H&E stains of both treated and untreated whole eyes showed no significant differences between groups (**Figure** **8**A). We quantified the corneal thickness and found no significant difference before and after drug treatment. Before treatment, the mean thickness was 72.8 ± 7.6 µm, and after 14 days was 72.5 ± 7.6 µm (n = 6, *P* = 0.94, unpaired two‐tailed *t*‐test). Closer inspection using confocal microscopy showed that the eyes treated with the BM/BX SDNs formulation did not adversely change the retinal layer structure (Figure 8B). Retinal layer thickness prior to treatment had a mean of 198.6 ± 19.7 µm and 14 days after treatment had a mean of 186.7 ± 13.9 µm (n = 7, *P* = 0.21, unpaired two‐tailed *t*‐test). Neither did thickness of cornea change during the treatment (Figure 8C). The drug treatment did not influence visual acuity, as indicated by the optomotor assessment (Figure 8D). Before treatment, the mean acuity was 0.39 ± 0.03 cyc/deg, and after 7 days was 0.36 ± 0.08 cyc/deg (n = 10, *P* = 0.35, unpaired two‐tailed *t*‐test). Together, these findings support the safety of the BM/BX SDNs.

## 3 Discussion

Nanocarriers have emerged as promising candidates for improving glaucoma management. Natarajan et. al. developed latanoprost‐encapsulated liposomes for sustained drug release and reducing IOP in a diseased nonhuman primate model.^[^ ^11^ ^]^ We previously reported dendrimer‐based nanogels for ocular drug delivery of antiglaucoma drugs, and the nanogels improved the therapeutic effect by elevating the cornea permeation.^[^ ^10^ ^]^ However, the successful application of nanocarriers has been hindered by the potential side effects aroused by the materials that make up the carriers and unsatisfied drug loading efficiency. Besides, the difficulties in scale‐up generation also restricted the clinical translation of nanoparticle‐based eyedrop formulations. In this study, we developed a flash nanoprecipitation method based on MIVM to prepare SDNs of antiglaucoma drugs. The SDNs are composed of only drugs, thereby avoiding potential side effects caused by carriers and the issue of low drug‐loading capacity. The successful fabrication of three SDNs using different hydrophobic antiglaucoma drugs BM, BX and the mixture of them proved the robustness of this method. More importantly, the size distribution of SDNs from three independent batch showed negligible variation, indicating excellent batch to batch consistency.

One of the major obstacles that limit the successful translation of nanoparticle formulations is the instability during long‐term storage. To address this issue, we adopted a commonly used strategy which is lyophilization to get rid of water from the formulation and allow long‐term storage of the formulation in a dry powder form. However, the process of lyophilization generates various stresses, which in turn may destabilize colloidal suspension of the nanoparticles. To protect the nanoparticles from freezing and desiccation stresses, lyoprotectants are added to the formulations. Sugars, such as trehalose, sucrose, glucose, sorbitol, and mannitol, are most reported lyoprotectants due to the advantages of low cost and abundant sources.^[^ ^28^, ^29^, ^30^ ^]^ Among them, trehalose, a natural disaccharide, has been verified to be safe and beneficial to the dry eye disease due to its bioprotective characteristics and activity against dehydration.^[^ ^25^, ^26^ ^]^ Therefore, we chose trehalose as lyoprotectant for BM/BX SDNs. The reconstituted BM/BX SDNs showed no significant difference with the freshly prepared BM/BX SDNs in both appearance and size distributions. Reconstitution of the eyedrop formulations before each administration may cause extra burden for the patients, thus it will be ideal if the reconstituted formulation remains stable for a few days. Our results suggested that the reconstituted BM/BX SDNs can keep stable in the refrigerator for at least 1 week. Hence, the BM/BX SDNs can be stored in lyophilized powder form, and easily reconstituted before administration with trehalose as lyoprotectant. The unspent eyedrop is stable for further use for a few days. Additionally, the BM/BX SDNs released BM and BX in a controlled manner because the drugs were released via particle dissolution and passive diffusion, with the diffusion rate primarily governed by the concentration gradient between the formulations and their surrounding environment. This gradient gradually decreased as the drugs were released. Importantly, the BM/BX SDNs demonstrated a longer release period compared to their hydrophilic counterparts, BT/BH. This extended release is likely due to the slower movement of hydrophobic BM and BX in an aqueous environment compared to hydrophilic BT and BH. Consequently, the BM/BX SDNs may enable a prolonged action time for the antiglaucoma drugs.

Biocompatibility and ocular tolerability are critical parameters in the development of ocular delivery systems. With the assistance of trehalose, the reconstituted BM/BX SDNs exhibited improved biocompatibility against HCE‐2 cells. HET‐CAM test, a sensitive, rapid, and cost‐effective method using incubated eggs, represents an efficient approach for detection of ocular corrosives and irritants.^[^ ^31^ ^]^ The HET‐CAM assay demonstrated that the BM/BX SDNs exhibited no signs of irritation upon applied to the CAM, indicating their non‐irritant properties. These characteristics make the BM/BX SDNs promising for clinic translation in the near future.

Unsatisfied cornea permeation caused by the poor affinity between the hydrophilic drugs and the lipophilic cell membrane has been one of the main obstacles that lead to low bioavailability of antiglaucoma drugs. Unlike small molecules, it is well documented that the nanoparticles can pass through biological membranes via transcytosis.^[^ ^32^, ^33^, ^34^ ^]^ Therefore, the BM/BX SDNs were expected to have better bioavailability than the commercially used BT and BH. To investigate the permeation capacity of the BM/BX SDNs, ex vivo cornea permeation was performed using an excised rabbit cornea to serve as the cornea barrier. Results revealed that the amount of BM and BX across the cornea was almost threefold higher than that of BT and BH. The results encouraged us to further evaluate the ocular permeation by in vivo experiments. Since it's difficult to label the BM/BX SDNs with fluorescent dye, we detected the drugs in the aqueous humor by LC‐MS and adopted MALDI‐IMS to directly detect the drugs in the eye tissues. MALDI‐IMS has become a widely used technique for a broad spectrum of analytes ranging from proteins, peptides, drugs, and their metabolites. This label‐free in situ technique makes possible to correlate molecular information with traditional histology by keeping the spatial localization information of the analytes after mass spectrometric measurement, and it has been used to detect the distribution of BM and BX in eyes previously.^[^ ^35^, ^36^, ^37^ ^]^ The significant enhancement of drugs in both aqueous humor and eye sections of rats was observed. It is noteworthy that different animal eyes were used for ex vivo and in vivo experiments to balance the requirements of experimental design and availability, recognizing that permeation coefficients vary between animal eyes.^[^ ^38^ ^]^ However, the primary focus of this study was to compare the permeation of BM/BX SDNs with their hydrophilic counterparts, BT and BH. The results from both ex vivo and in vivo permeation experiments collectively demonstrate that BM/BX SDNs can significantly enhance the permeation of antiglaucoma drugs.

The elevated IOP In the glaucomatous eyes was ascribed to the build‐up of aqueous humor, which can be alleviated by increasing the outflow and/or suppressing the production of the fluid. The mechanism of BX is to reduce the generation of aqueous humor, while BM can simultaneously promote the outflow and hinder the production of aqueous humor. Usually, separate use of these two drugs in safe doses is not efficient enough and it was reported the combination treatment of them achieved synergetic effect in IOP reduction.^[^ ^39^ ^]^ However, co‐delivery of different drugs in one nanoparticle‐based formulation remains challenging. The manner the SDNs were prepared made it easy to combine different drugs because we only need to dissolve the drugs in one solution with pre‐determined ratio before the vortex mixing. Incorporating the merits of combination therapy and enhanced bioavailability, the BM/BX SDNs were supposed to be promising in obtaining better IOP control. This was preliminarily confirmed by the enhanced IOP reduction and longer action time in normotensive rats. Subsequently, the IOP lowering effect of the BM/BX SDNs was further investigated using A1 cKO mice as a glaucomatous model. The A1 cKO mice exhibit consistent ocular hypertensive starting at one month of age, making it an ideal model to longitudinally monitor the drug effects on controlling IOP.^[^ ^40^, ^41^, ^42^ ^]^ We demonstrated that A1 cKO mice exhibit consistent IOP elevation starting from ≈20‐days of age.^[^ ^43^ ^]^ Therefore, A1 cKO mice is an ideal model for short‐ and long‐term study of the curative effect of antiglaucoma medicines. Our results indicated that the BM/BX SDNs showed consistent IOP lowering effect than their hydrophilic counterparts. Repeated dosing of BM/BX SDNs every other day resulted in consistent IOP reduction, in contrast to the IOP fluctuations of A1 cKO mice treated with non‐formulated BM/BX. Our studies thus support the notion that the SDN formulated drug could control IOP consistently with missed dosages when the prescribed daily dose is used for patients.

## 4 Conclusion

In this study, we successfully developed a novel method for scalable fabrication of efficient SDN‐based eye drop formulations using hydrophobic antiglaucoma drugs. This method enables preparation of uniform BM SDNs, BX SDNs and BM/BX SDNs with excellent batch‐to‐batch consistency. With trehalose as cryoprotectant, the BM/BX SDNs can be well reconstituted after lyophilization and the reconstituted formulations exhibited good colloidal stability, prolonged release period, enhanced cytocompatibility and non‐irritant properties. More importantly, the BM/BX SDNs enhanced the ocular permeation by about threefold, revealed by both ex vivo and in vivo experiments. Benefitting from the promoted bioavailability, significant improvement of IOP lowering effect was observed on both normal rats and glaucoma mice, and topical administration of the BM/BX SDNs every other day can keep the IOP in a stable and low level. Incorporating the merits of scalable and low‐cost fabrication, as well as significantly enhanced therapeutic effect, the proposed strategy and the obtained BM/BX SDNs are promising for development of translational eyedrop formulations for improved glaucoma management.

## 5 Experimental Section

### Materials

Brimonidine (BM, 98%) was purchased from Thermo Scientific (Waltham, MA). Betaxolol (BX, 98%) was purchased from Medchemexpress LLC (Monmouth Junction, NJ). Trehalose (98%) was purchased from TCI America (Portland, OR). Cell proliferation reagent CCK‐8 was purchased from Apexbio Technology LLC (Boston, MA). White Leghorn chicken eggs (SPF fertile) were acquired from AVS Bio (Norwich, CT). Fresh rabbit whole eyes were purchased from Pel‐Freeze Biologicals (Rogers, AR). Cell culture medium Keratinocyte SFM for human corneal epithelial cells (HCE‐2, ATCC, Manassas, VA) was purchased from Thermo Fisher Scientific (Waltham, MA).

### Preparation of SDNs

BM and BX were dissolved in methanol with a concentration of 0.4 and 1 mg mL^−1^ respectively. The drug solution was introduced to one inlet of MIVM and water was introduced to the remaining three inlets. Then the four streams were mixed in the MIVM with a flow rate of 40 mL min^−1^. After removal of methanol by rotary evaporation, the BM/BX SDNs were obtained. Subsequently, trehalose was added to the solution and the solution was freeze‐dried. The yielded pale‐yellow powder was stored at room temperature and PBS (pH = 7.4) was added to reconstitute it before use. Following the same procedures, BM SDNs and BX SDNs were prepared by replacing the BM/BX mixed solution with BM solution or BX solution.

### Characterization—Transmission Electron Microscopy (TEM)

TEM images were obtained on a JEM‐2200FS microscope (JEOL, Japan) after the samples were negatively stained by sodium phosphotungstate. Generally, a 3 µL droplet of nanoparticles solution with a concentration of 1 mg mL^−1^ was dropped onto a copper grid (300 mesh) coated with a carbon film, then the excess liquid was wicked off and the grids were immediately placed onto individual droplets of freshly prepared and filtered, 2 wt% aqueous sodium phosphotungstate. After 2 min, excess stain was removed, and the grids were allowed to dry thoroughly.

### Characterization—Dynamic Light Scattering (DLS)

Size distribution and zeta potentials of the nanoparticles were characterized by DLS using a Lab Red zetasizer (Malvern, UK) with a 632.8 nm laser light set at a scattering angle of 90°. Each measurement was performed in triplicate, and the results were processed with ZS XPLORER version 1.02.

### Characterization—Liquid Chromatography–Mass Spectrometry (LC‐MS)

For the drug release and cornea permeation experiments, the concentrations of BM and BX were measured by using a LC‐MS. A LCMS‐2020 single quadrupole mass spectrometry (Shimadzu, Japan) coupled with a LC‐2040C ultra‐high performance liquid chromatography system (Shimadzu, Japan) was used. Analytes were separated on Synergi Hydro‐RP 80 Å column (2 × 150 mm, 4 µm) using water and acetonitrile containing 0.005% formic acid (v/v) as mobile phase at a flow rate of 0.3 mL min^−1^.

### Characterization—Matrix‐Assisted Laser Desorption Ionization Imaging Mass Spectrometry (MALDI‐IMS)

MALDI‐IMS was performed on a MALDI‐LTQ‐Orbitrap XL mass spectrometer (Thermo Scientific, San Jose, CA) equipped with a nitrogen laser emitting radiation at 337 nm with a repetition rate of 60 Hz, and a focal spot size of ≈100 µm × 80 µm. *α*‐Cyano‐hydroxycinnamic acid was used as matrix and applied to the slides using an air‐brush. The m/z = 291.836 (theoretical [M_BM_+H]^+^ = 292.0198) and m/z = 308.043 (theoretical [M_BX_+H]^+^ = 308.2225) were selected to detect BM and BX respectively. The mass resolving power was set to 30 000 for all MS experiments.

### Cytotoxicity Assay

HCE‐2 cells were cultured at 37 °C in Keratinocyte SFM (Cat# 1700542) medium supplemented with EGF human recombinant and Bovine Pituitary Extract in a 5% CO_2_ incubator. The culture flasks were precoated with solution of Collagen I (rat tail, C3867) and Bovine Serum Albumin (A8412) both purchased from Sigma, St. Louis MO. After the cells reach a confluence of 70–80%, they were detached by trypsin to make a cell suspension. Subsequently, the cells were seeded into a 96‐well plate with a density of 2 × 104 per well and cultured overnight. Then trehalose, BM/BX SDNs with or without trehalose with various concentrations were added to the medium. After incubation for 24 h, the medium was replaced with 100 µL of fresh medium and 10 µL of CCK‐8 was added to each well, followed by incubation at 37 °C in the 5% CO_2_ incubator for 3 h. The absorbance of the medium in each well at 570 nm were measured by a Synergy 2 microplate reader (BioTek, US), and the relative viability of the cells were calculated by comparing the absorbance of the treated well to that of the untreated wells. The data were presented as mean ± SD (n = 5).

### Irritation Assessment of SDNs by the HET‐CAM Assay

The HET‐CAM assay was a sensitive in vitro assay that uses fertilized hen eggs. The CAM membrane was highly vascularized, which mimics the mucosal and subcutaneous tissue.^[^ ^31^ ^]^

Fertile SPF White Leghorn chicken eggs were kept at room temperature for 4–6 h post‐delivery from AVS Bio (Norwich, CT). Subsequently, the eggs were incubated horizontally in a 60% humidified atmosphere for three days. Post‐incubation, the eggs were carefully opened, and the embryos with viable heartbeat were transferred into sterilized weigh boats, with a small portion of eggshell to facilitate normal development. The viable chick embryos in weigh boats inside a specialized humidified chamber were incubated at 37.5 °C for seven days. On day 7, the formulation was tested for irritation potential on CAMs.

Briefly, for testing the formulations a sterilized 10‐mm diameter poly‐band ring was positioned on a region of the chick embryo devoid of major blood vessels. Within these rings, 20 µL of standard controls and formulations—Normal saline, 0.1 n NaOH, or BM/BX SDNs (with 0.2% w/v BM and 0.5% w/v BX) were administered and monitored for 300 s. The CAM was meticulously inspected for the time to appearance of hemorrhage (H), vascular lysis (L) and coagulation (C) under 1× magnification using a Leica Stereo Zoom S8 AP0 microscope, operated with Leica software (Leica Microsystems Inc, Buffalo Grove, IL). The experiment was performed in triplicated in three different viable CAM.

The HET‐CAM irritation score and Irritation category of the SDNs were calculated and classified based on Protocol No. 96: ″ Hen's Egg Test on the Chorioallantoic Membrane (HET‐CAM) provided by EURL ECVAM dataset on alternative methods to animal experimentation (DB‐ALM) guidelines.^[^ ^44^ ^]^ Accordingly, the time noted in seconds were applied to the predictive model formula to calculated the irritation score (IS) = (301 – H)*5/300 + (301 – L)*7/300 + (301 – C)*9/300.

### In Vitro Drug Release

Typically, BT/BH solutions or reconstituted BM/BX SDNs in PBS (pH = 7.4) with equal BM and BX concentrations were encapsulated in a Pur‐A‐Lyzer dialysis tube (MWCO 6000 Da). Then the tube was immersed in 5 mL of PBS and the set was incubated at 37 °C with shaking (100 rpm). At predetermined intervals, 100 µL of the release medium was withdrawn and replaced with equal volume of fresh medium. The concentrations of BM and BX in the samples were analyzed using LC‐MS and the cumulative release ratios of BT and BH from the BT/BH solution as well as BM and BX from BM/BX SDNs were calculated. The experiments were carried out in triplicate.

### Ex Vivo Cornea Permeation

Cornea excised from fresh rabbit eye was mounted between the donor chamber and receptor chamber of a Franz diffusion cell system. Then BT/BH solutions or reconstituted BM/BX SDNs in PBS (pH = 7.4) with equal BM and BX concentrations were added to the donor chamber and the receptor chamber was filled up with PBS. The set was incubated at 37 °C. At predetermined intervals up to 12 h, an aliquot of 100 µL was withdrawn from the receptor chamber and analyzed by LC‐MS. An equal volume of fresh PBS was supplemented to the receptor chamber following each sampling.

### In Vivo Permeation and Biodistribution

Wistar female rats (5 months old) were used and housed in individually ventilated cage at Missouri University of Science and Technology (MST). The rats were kept under 12 h light/12 h dark circle and fed with standard rodent diet and tap water. All experiments performed on the rats comply with the protocol which was approved by the institutional animal care and use committees of MST.

Given that this study mainly focuses on comparing the permeation and therapeutic effects of BM/BX SDNs with their hydrophilic counterparts, BT and BH, these two formulations were administered to each eye of the same animals, respectively, to facilitate this comparison and minimize the impact of individual differences. Briefly, the right and left eyes of rats were topically treated with BM/BX SDNs (2 × 5 µL per eye, 0.2% w/v BM and 0.5% w/v BX) and BT/BH solutions (2 × 5 µL per eye, equal to 0.2% w/v BM and 0.5% w/v BX) respectively. After 4 h, the rats were sacrificed by CO_2_. For cornea permeation studies, aqueous humors were withdrawn and precipitated in methanol to remove proteins. The supernatants were analyzed by LC‐MS to calculate the concentration of drugs in the aqueous humors.

For biodistribution studies, the eyeballs were excised and washed with PBS, followed by freezing in isopentane cooled with liquid nitrogen. Subsequently, the frozen eyeballs were embedded in 2% w/v carboxymethylcellulose, frozen in isopentane cooled with liquid nitrogen and stored at −80 °C before cryo‐sectioning. The frozen blocks were equilibrated at −20 °C on a cryostat and then sectioned into 10‐µm‐thick slices. The slides were applied to the surface of a 20‐Ω indium/tin oxide‐coated glass slide, coated with matrix, and observed by MALDI‐IMS.

### In Vivo IOP Reduction Evaluation on Normotensive Rats

Brown Norway female rats (5 months old, n = 8) were used for IOP reduction evaluation. Before the treatment, IOP of the rats were measured at 9 am for three successive days using a ICare TONOLAB tonometer to obtain the baseline. On fourth day, the right and left eyes of rats were topically treated with BM/BX SDNs (2 × 5 µL per eye, 0.2% w/v BM and 0.5% w/v BX) and BT/BH solutions (2 × 5 µL per eye, equal to 0.2% w/v BM and 0.5% w/v BX) respectively at 9 am. Then the IOP of both eyes were measured at 6, 24, 30, 48, 72, 96, 120 h post treatment. The concentrations of drugs and dosages were defined according to previous studies.^[^ ^10^, ^39^ ^]^

Before the treatment, IOP of the rats were measured at 9 am for three successive days to obtain the baseline. Then every other day, the right and left eyes of rats were topically treated with BM/BX SDNs (2 × 5 µL per eye, 0.2% w/v BM and 0.5% w/v BX) and BT/BH solutions (2 × 5 µL per eye, equal to 0.2% w/v BM and 0.5% w/v BX) respectively at 9 am. Totally three successive doses were administered and the IOP was monitored for 7 days. During the treatment, the IOP of both eyes were measured at 9 am and 3 pm every day.

### In Vivo Evaluations on Glaucomatous Mice

Two to twelve‐month‐old A1 cKO mice, a genetic model for open angle glaucoma, was used. Drug treatment regimens were the same as described above for normotensive rats, except that the dosages for mice were 2 × 2 µL for each eye. IOP of the A1 cKO mice was monitored to evaluate therapeutic effect of the formulations.

Mouse visual acuity was monitored by the optomotor test before and after treatment of three successive doses (7 days). The free‐moving animal was placed on a stationary round platform in the middle of four LCD monitors. A sine wave grating was made to move across all four monitors, and the movement of the animal's head in‐concert with the drifting grating was scored “seen”; The highest spatial frequency “seen” was defined as the animal's visual acuity. The optomotor test permits one to test each eye of a mouse separately by reversing the stimulus's direction of motion, so that one eye can be tested with experimental manipulation and use the other eye of the same mouse as control.^[^ ^45^, ^46^, ^47^ ^]^ By the end of experiments, mice were sacrificed, and eyes dissected for confocal imaging to examine eye and retinal morphology.^[^ ^48^ ^]^ In brief, mice were euthanized with Euthasol (15.6 mg mL^−1^; Virbac) and perfused them with 4% paraformaldehyde (PFA) in PBS.^[^ ^49^, ^50^ ^]^ Eyecups were dissected and post‐fixed in PFA for 30 min. Cryo‐sections were prepared and slides were cover‐slipped with Vectashield mounting medium (Vector Laboratories Inc.).^[^ ^46^, ^47^, ^48^, ^49^, ^50^ ^]^ Confocal microscopy was performed using a Zeiss LSM 800 microscope (Carl Zeiss AG).^[^ ^46^, ^47^, ^48^, ^49^, ^50^ ^]^

### Statistical Analysis

Data were presented as mean ± standard deviation (SD) or standard error (SE). The plotting and statistical analysis were performed using GraphPad Prism 8. Student's *t*‐test was conducted to compare the statistical difference between paired group. Statistical significance was indicated as ^*^ *p* < 0.05; ^**^ *p* < 0.01 etc.

## Conflict of Interest

The authors declare no conflict of interest.

## Supporting information

Supporting Information

## Data Availability

The data that support the findings of this study are available from the corresponding author upon reasonable request.
