# Supplementary material for: Consistent Intraocular Pressure Reduction by Solid Drug Nanoparticles in Fixed Combinations for Glaucoma Therapy
Source: Adv Sci (Weinh). 2024 Jun 14;11(31):2401648. doi: 10.1002/advs.202401648 (PMC11336906; doi:10.1002/advs.202401648)
Supplement: Supplementary file 1 — Supporting Information [file ADVS-11-2401648-s001.docx]

**Supplementary Information**

**Consistent Intraocular Pressure Reduction by Solid Drug Nanoparticles in Fixed Combinations for Glaucoma Therapy**

Da Huang^1,2^, Pedro Norat^3^, Lin Qi^2^, Anna Chernatynskaya^2^, James D. Cole^3, †^, Vimalin Jeyalatha Mani^2^, Lei Xu^2^, Xiaorong Liu^3, 4, 5^ *, Hu Yang^2^ *

^1^College of Biological Science and Engineering, Fuzhou University, Fuzhou, Fujian 350108, China

^2^Linda and Bipin Doshi Department of Chemical and Biochemical Engineering, Missouri University of Science and Technology, Rolla, MO 65409, United States

^3^Department of Biology, University of Virginia, Charlottesville, VA 22904, United States

^4^Department of Psychology, University of Virginia, Charlottesville, VA 22904, United States

^5^Program in Fundamental Neuroscience, University of Virginia, Charlottesville, VA 22904, United States

*Corresponding authors. xln8@virginia.edu (XL) and huyang@mst.edu (HY)

^†^ Current address: Department of Psychology, West Virginia University, Morgantown, WV 26506


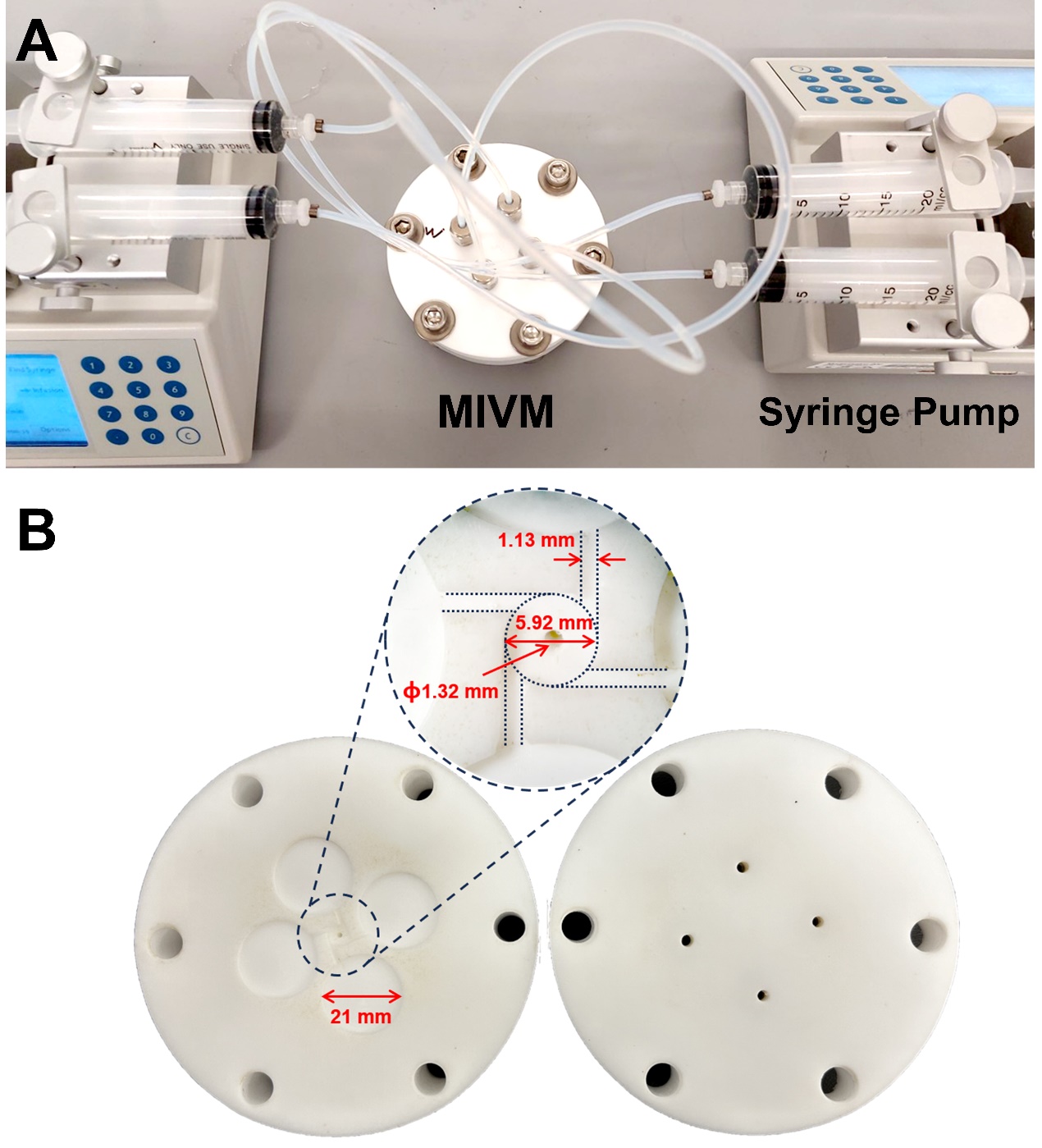


**Figure S1.** (A) Photo of MIVM-based nanoparticle generation system and (B) inner design of the MIVM.

**
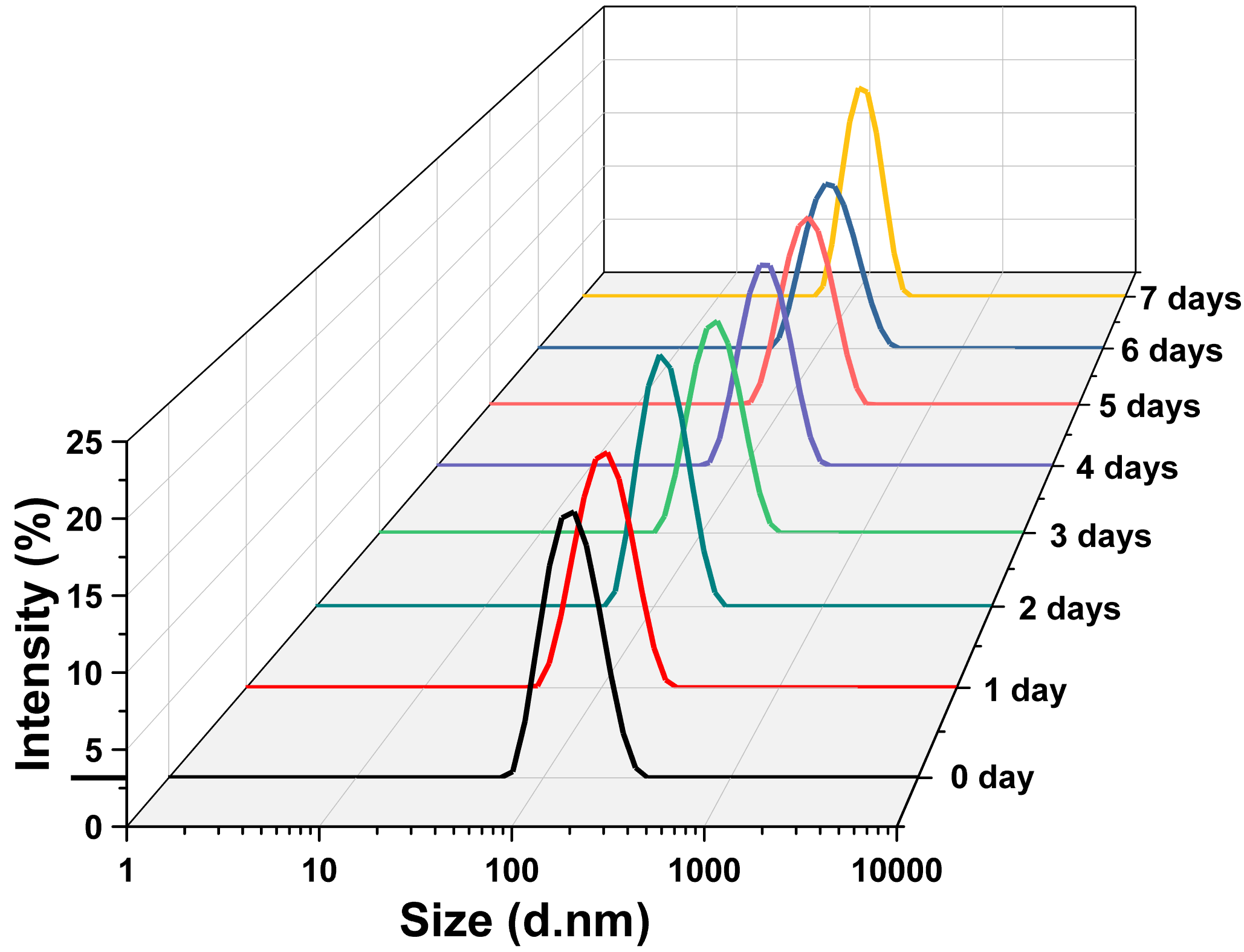
Figure S2.** Size distributions of the redispersed BM/BX SDNs solution after being stored at 4 °C for different days.

**
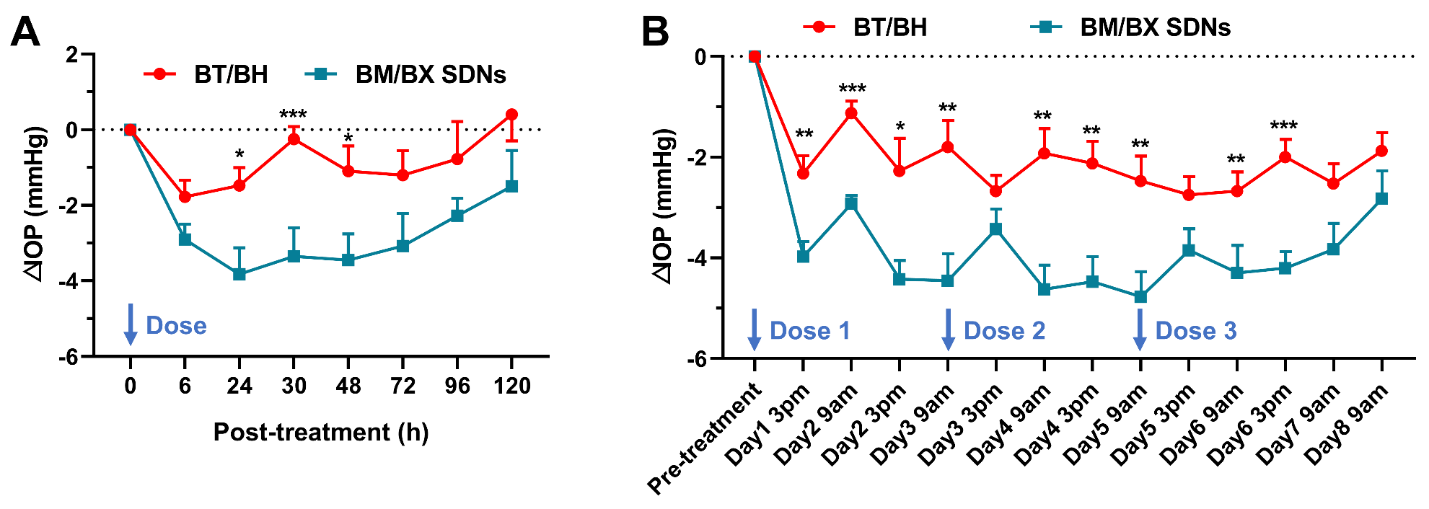
**

**Figure S3.** IOP reduction of normotensive rats treated with (A) a single dose or (B) three successive doses of BT/BH or BM/BX SDNs (0.2% w/v BM, 0.5% w/v BX, 2*5 μL per eye). Statistical analysis was performed by unpaired Student’s t-test (two-tailed), and data were expressed as mean ± SE (n = 6), * *P* < 0.05, ** *P* < 0.01, *** *P* < 0.005.
